# Development of a Novel *mcr-6* to *mcr-9* Multiplex PCR and Assessment of *mcr-1* to *mcr-9* Occurrence in Colistin-Resistant *Salmonella enterica* Isolates From Environment, Feed, Animals and Food (2011–2018) in Germany

**DOI:** 10.3389/fmicb.2020.00080

**Published:** 2020-02-04

**Authors:** Maria Borowiak, Beatrice Baumann, Jennie Fischer, Katharina Thomas, Carlus Deneke, Jens Andre Hammerl, Istvan Szabo, Burkhard Malorny

**Affiliations:** Department Biological Safety, German Federal Institute for Risk Assessment (BfR), Berlin, Germany

**Keywords:** *Salmonella*, colistin resistance, *mcr* genes, multiplex PCR, multidrug resistance

## Abstract

The polymyxin antibiotic colistin has been used in decades for treatment and prevention of infectious diseases in livestock. Nowadays, it is even considered as last-line treatment option for severe human infections caused by multidrug- and carbapenem-resistant Gram-negative bacteria. Therefore, the discovery of plasmid-mediated mobile colistin resistance (*mcr*) genes raised major public health concern. The aim of our study was to analyze colistin-resistant *Salmonella enterica* strains from animals, food, feed and the environment collected at the National Reference Laboratory for *Salmonella* in Germany on the presence of *mcr-1* to *mcr-9* genes. Altogether 407 colistin-resistant (MIC >2 mg/L) *Salmonella* isolates received between 2011 and 2018 were selected and screened by PCR using a published *mcr-1* to *mcr-5* as well as a newly developed *mcr-6* to *mcr-9* multiplex PCR protocol. 254 of 407 (62.4%) isolates harbored either *mcr-1* (*n* = 175), *mcr-4* (*n* = 53), *mcr-5* (*n* = 18) or *mcr-1* and *mcr-9* (*n* = 8). The number of *mcr*-positive isolates ranged from 19 (2017) to 64 (2012) per year. WGS revealed that none of our isolates harbored the *mcr-9.1* gene. Instead, two novel *mcr-9* variants were observed, which both were affected by frameshift mutations and are probably non-functional. The *mcr*-harboring isolates were mainly derived from animals (77.2%) or food (20.1%) and could be assigned to ten different *Salmonella* serovars. Many of the isolates were multidrug-resistant. Co-occurrence of *mcr-1* and AmpC or ESBL genes was observed in eight isolates. Our findings suggest that *mcr* genes are widely spread among colistin-resistant *Salmonella* isolates from livestock and food in Germany. Potential transfer of *mcr*-harboring isolates along the food chain has to be considered critically.

## Introduction

Non-typhoidal *Salmonella* (*S.*) *enterica* subsp. *enterica* are zoonotic pathogens which can cause severe human infections (salmonellosis) through the consumption of contaminated food. Spread of antibiotic resistance in *S. enterica* might impair successful antibiotic treatment of salmonellosis in patients where it is indispensable (Michael and Schwarz, 2016).

The polypeptide antibiotic colistin belongs to the group of polymyxins and has been used for decades in the animal production for treatment and prevention of infectious diseases (EMA, 2016). Comparing 30 European countries, Germany had the sixth highest polymyxin sale for food-producing species in 2016 (EMA and ESVAC, 2017). Especially colistin is frequently used in pig, cattle, sheep and poultry production, mainly for the treatment of gut infections caused by *S. enterica* and *Escherichia* (*E.*) *coli* (Emmerich and Drees, 2016). In human medicine, colistin played a minor role for long time, due to high side effects and the availability of better alternatives for antimicrobial treatment (Yu et al., 2015). However, in the last years, colistin was rediscovered for last-line treatment of severe human infections caused by multidrug- and carbapenem-resistant Gram-negative pathogens and is now considered as a critically important antimicrobial for human medicine (Falagas et al., 2005; Grégoire et al., 2017; World Health Organization [WHO], 2017). Therefore, the first discovery of a plasmid-encoded mobile colistin resistance gene (*mcr-1*) in 2015 in *E. coli* isolated from livestock animals, meat and patients as well as in *Klebsiella pneumoniae* from clinical settings in China raised major public health concern (Liu et al., 2016; Sun et al., 2018). Extensive follow-up screenings identified *Moraxella* spp. as potential origin of *mcr-1* (Kieffer et al., 2017) and revealed that this gene can be found in various pathogenic and commensal Enterobacteriaceae from the environment, wildlife, livestock, food and patients worldwide (Sun et al., 2018; Lima et al., 2019). Due to the high prevalence, it is thought that food-producing animals are most likely the origin for *mcr-1* mediated colistin resistance (Nordmann and Poirel, 2016; Poirel and Nordmann, 2016). These findings and the reclassification of colistin in human medicine led to a reassessment of colistin by the European Medicines Agency (EMA) suggesting to reduce the use of colistin in livestock production as much as possible (EMA, 2016).

After the detection of *mcr-1*, seven additional *mcr* genes (*mcr-2* to *mcr-8*) were described (Xavier et al., 2016; Abuoun et al., 2017; Borowiak et al., 2017a; Carattoli et al., 2017; Yin et al., 2017; Wang et al., 2018; Yang et al., 2018). Recently, the inducible *mcr-9* gene was added to the increasing number of *mcr* genes (Carroll et al., 2019). These results further enhanced public health concerns related to the emergence, persistence and spread of mobile colistin resistance and reinforced the call for ongoing extensive screenings in Enterobacteriaceae.

In this study we developed a gel-based multiplex PCR protocol which detects *mcr-6, mcr-7, mcr-8*, and *mcr-9* for rapid screening purposes of bacterial isolates. We applied the novel *mcr-6* to *mcr-9* assay in combination with a previously published multiplex PCR for detection of *mcr-1* to *mcr-5* (Rebelo et al., 2018) on 407 colistin-resistant *S. enterica* isolates from the strain collection of the German National Reference Laboratory for *Salmonella*. These isolates were collected between 2011 and 2018 in the frame of routine diagnostics and national surveillance and monitoring programs and originated from livestock, animals, food, feed and the environment in Germany.

## Materials and Methods

### Strain Selection and DNA Extraction

PCR screening was performed on 407 initially phenotypically colistin-resistant (MIC >2 mg/L) *S. enterica* isolates (with exception of serogroup D) obtained from livestock, animals, food, feed and the environment, collected in the frame of routine diagnostics as well as national monitoring and surveillance programs in the years 2011 to 2018 in Germany at the National Reference Laboratory for the Analysis and Testing of Zoonoses (NRL *Salmonella*). All strains selected have no epidemiological link (neither isolated at the same place, time, animal or food). All isolates were routinely subjected to serotyping by slide agglutination with antisera raised against O-, H1- and H2- antigens and MIC testing as described before (Borowiak et al., 2017a). For PCR screening, isolates were cultivated from stock cultures by inoculating liquid LB medium and subsequent incubation under shaking conditions (250 × rpm) for 16 h at 37°C. Thermal cell lysis preparations were produced as previously described (Borowiak et al., 2017a). DNA suitability for PCR amplification was confirmed by a standard PCR protocol for amplification of the housekeeping gene *hemD* using the primers recommended on Enterobase^1^. The *hemD* PCR was prepared in 25 μL including 12.5 μL 2x DreamTaq Green PCR Master Mix (Thermo Scientific, Vilnius, Lithuania), 0.5 μL of each 10 μM primer dilution, 9.5 μL PCR grade water and 2 μL of thermal cell lysis preparation as template DNA and carried out as follows: initial denaturation for 3 min at 95°C, 35 cycles denaturation for 30 s at 95°C, primer annealing for 30 s at 55°C and elongation for 30 s at 72°C followed by a final elongation step for 10 min at 72°C.

### Multiplex PCR Methods

#### Controls

Positive controls for *mcr-1*, *mcr-4*, *mcr-5*, and *mcr-9* were obtained using in-house strains *S.* Paratyphi B *d*T+ 08-00436 (*mcr-1.1*; BioSample: SAMN06645218), monophasic *S.* Typhimurium 15-SA02945 (*mcr-4.2*; BioSample: SAMEA104398406), *S.* Paratyphi B *d*T+ 13-SA01718 (*mcr-5.1*; BioSample: SAMN07420414) and *S.* Infantis 15-SA01028 (*mcr-9.1*; BioSample: SAMN08475067). Control strains for *mcr-2* and *mcr-3* were kindly provided by the National Food Institute, Technical University of Denmark (Copenhagen).

For *mcr-6* to *mcr-8* positive controls, DNA was synthesized (Integrated DNA Technologies, Leuven, Belgium) and subjected to PCR and cloning. Following gene regions were selected for DNA synthesis: *mcr-6.1* (GenBank: NG_055781.1, bases 557-896), *mcr-7.1* (GenBank: NG_056413.1:101-1720 bases 190-780), and *mcr-8.1* (GenBank: NG_061399.1:101-1798, bases 630-1566).

All primer used in this study were synthesized by Eurogentec (Seraing, Belgium). PCR reactions for cloning were prepared in 50 μL volumes comprised of 2 μL synthetic DNA (final amount: 0.1 ng), 5 μL of 10× PCR Rxn Buffer, 1.5 μL of 50 mM MgCl_2_, 5 μL of 200 μM dNTP Mix (prepared from 100 mM dNTP solutions), 0.4 μL of Platinum^®^ Taq DNA Polymerase (Invitrogen, Carlsbad, CA, United States), 5 μL of the respective 10 μM primer dilutions, and 26.1 μL PCR grade water. The primer used in the respective cloning PCR reactions were the same as used in the multiplex PCR protocol (Table 1). PCR was carried out in following steps: initial denaturation for 5 min at 95°C (all), 25 cycles denaturation for 30 s at 95°C (all), primer annealing for 30 s at 52°C (*mcr-6*), 50°C (*mcr-7*) or 53°C (*mcr-8*), elongation at 72°C for 20 s (*mcr-6*), 35 s (*mcr-7*) or 55 s (*mcr-8*) followed by a final elongation step for 5 min at 72°C (all). Freshly prepared PCR products were cloned into pCR^TM^ 2.1-TOPO^®^ vectors and transferred in One Shot^®^ chemically competent *E. coli* TOP10F’ cells from the TOPO^®^ TA Cloning^®^ Kit (Invitrogen) following manufactures instructions. Positive transformants were selected on LB agar supplemented with 100 mg/L ampicillin (Sigma, Darmstadt, Germany) and confirmed by PCR. Plasmid DNA of transformants TOP10F’ + pCR2.1-mcr-6, TOP10F’ + pCR2.1-mcr-7, and TOP10F’ + pCR2.1-mcr-8 was extracted using the Invisorb^®^ Spin Plasmid Mini Two Kit (Stratec, Berlin, Germany) and the insert sequence was confirmed by Sanger sequencing.

**TABLE 1:** Primer and PCR products for the *mcr-6* to *mcr-9* multiplex PCR assay.

Primer name	Primer sequence	Product length [bp]
mcr-6_mp_fw	5′-AGCTATGTCAATCCCGTGAT-3′	252
mcr-6_mp_rev	5′-ATTGGCTAGGTTGTCAATC-3′	
mcr-7_mp_fw	5′-GCCCTTCTTTTCGTTGTT-3′	551
mcr-7_mp_rev	5′-GGTTGGTCTCTTTCTCGT-3′	
mcr-8_mp_fw	5′-TCAACAATTCTACAAAGCGTG-3′	856
mcr-8_mp_rev	5′-AATGCTGCGCGAATGAAG-3′	
mcr-9_mp_fw	5′-TTCCCTTTGTTCTGGTTG-3′	1011
mcr-9_mp_rev	5′-GCAGGTAATAAGTCGGTC-3′	

#### Multiplex *mcr-1* to *mcr-5* PCR Method

PCR screening for *mcr-1* to *mcr-5* was carried out using the multiplex PCR protocol published by Rebelo et al. (2018) with the deviation that the CLR5-F and CLR5-R primers (Liu et al., 2016) were used for amplification of *mcr-1* resulting in a PCR product size of 309 bp.

#### Multiplex *mcr-6* to *mcr-9* PCR Method Development

**FIGURE 1:**
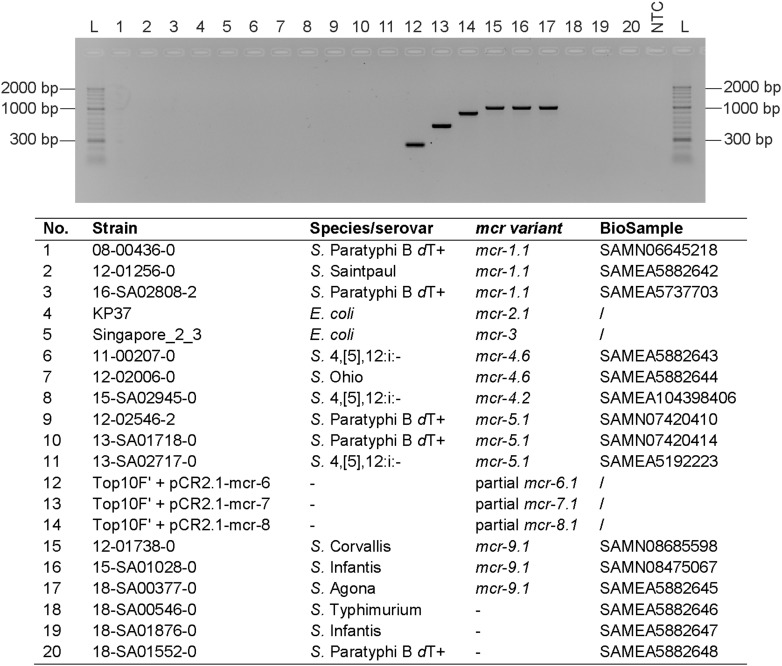
*mcr-6* to *mcr-9* multiplex PCR. The PCR was tested on DNA extractions of selected strains. PCR grade water was used as negative control (NTC) and HyperLadderII (Bioline) was used as molecular weight marker (L). The PCR products were electrophoretically separated on a 1.5% agarose gel (Bioline) supplemented with Midori Green (NIPPON Genetics Europe, Düren, Germany).

Four primer pairs, one for each *mcr-6* to *mcr-9* gene, were designed in CLC Genomics Workbench 9.5.2 (Qiagen, Hilden, Germany) and subsequently adapted manually. The primer pairs were developed to amplify the four target genes, with a size ranging from 252 to 1011 bp. This allows the separation and visualization of PCR products on agarose gels. The *mcr-6* to *mcr-9* multiplex PCR was carried out in 25 μL PCR reactions comprised of 12.5 μL 2x DreamTaq Green PCR Master Mix, 0.5 μL of each 10 μM primer dilution (Table 1), 6.5 μL PCR grade water and 2 μL of thermal cell lysis preparation as template DNA. The PCR conditions were optimized as follows: initial denaturation for 3 min at 95°C, 30 cycles denaturation for 30 s at 95°C, primer annealing for 30 s at 55°C and elongation for 60 s at 72°C followed by a final elongation step for 10 min at 72°C. PCR products were electrophoretically separated on 1.5% agarose gels (Bioline, London, United Kingdom). The novel *mcr-6* to *mcr-9* multiplex protocol, which is *in silico* compatible with all known *mcr-6* to *mcr-9* variants (*mcr-6.1*, *mcr-7.1*, *mcr-8.1*, *mcr-8.2*, and *mcr-9.1*), was initially tested on different *mcr-*positive as well as *mcr*-negative control strains previously identified by whole-genome sequencing (Figure 1).

### Whole-Genome Sequencing (WGS)

Non-serotypeable *mcr*-positive isolates, isolates showing phenotypic resistance to 3^rd^ generation cephalosporins or isolates harboring the recently detected *mcr-9* gene were subjected to WGS. DNA was extracted as previously described (Borowiak et al., 2017a), sequencing libraries were either prepared using the Nextera XT or the Nextera DNA Flex Kit (Illumina, San Diego, CA, United States) and sequencing was performed on a MiSeq or NextSeq benchtop sequencer (Illumina). Raw reads were trimmed using fastp v0.19.5 (Chen et al., 2018) and *de novo* assembled using unicycler v0.4.4 (Wick et al., 2017). Subsequently, the serovar was predicted from assembled sequencing data using SISTR (Yoshida et al., 2016). MLST2.0, ResFinder3.2 and PlasmidFinder2.1 provided by the Center of Genomic Epidemiology at DTU^2^ were further used to characterize the isolates. Whole-genome SNP analysis and clustering of isolates were performed as previously described (Borowiak et al., 2017a). As references, NCBI reference sequences NZ_CP026976.1 (*S.* Heidelberg) and NZ_CP019649 (*S.* Typhimurium) were used.

## Results

### Multiplex *mcr-6* to *mcr-9* PCR Development and Testing

Since *mcr-6*, *mcr-7*, and *mcr-8* isolates were, to our knowledge, not yet detected in Europe and the access of isolates was not possible, we cloned fragments of the particular genes into pCR^TM^ 2.1-TOPO^®^ vectors and transformed them into *E. coli* TOP10F’ cells. Thermal cell lysis preparations were subsequently used as positive controls. For *mcr-9*, three *mcr-9.1* positive isolates previously identified by routine WGS analysis were selected as control strains. For each *mcr-6* to *mcr-9* control the multiplex protocol amplified a PCR product forming a distinct band with the expected size after agarose gel electrophoresis. Primer pairs of the particular *mcr* genes did not interfere with any other *mcr* gene present in selected *mcr-1-* to *mcr-5*-positive strains (Figure 1).

### Distribution of *mcr* Genes in German Colistin-Resistant *Salmonella* Isolates

For PCR screening of colistin-resistant *Salmonella* isolates the *mcr-1* to *mcr-5* multiplex protocol published by Rebelo et al. (2018) as well as a newly developed multiplex PCR protocol for *mcr-6* to *mcr-9* was used. Results revealed that 254 out of 407 tested *Salmonella* isolates carried either *mcr-1* (175 isolates), *mcr-4* (53 isolates), *mcr-5* (18 isolates) or *mcr-1* and *mcr-9* (8 isolates) (Table 2). Other *mcr* genes (*mcr-2*, *mcr-3*, *mcr-6*, *mcr-7*, and *mcr-8*) were not detected. The number of *mcr*-positive isolates ranged from 19 to 33 isolates per year with the exception of 2012 (detection of 64 *mcr*-positive isolates). The *mcr-1*- and *mcr-4*-positive isolates were observed in every year, while *mcr-5*-harboring isolates were only detected in 2011 to 2013 and 2016, and *mcr-1*- and *mcr-9*-positive isolates only in 2013 to 2015 (Supplementary Table S1). The *mcr*-positive *Salmonella* isolates belonged to various serovars and were derived from different isolation sources. Most *mcr*-harboring *Salmonella* isolates were associated with livestock (77.2%, mainly pig and poultry). However, 20.1% of the isolates were derived from food products (including beef, pork, poultry meat as well as minced meat and meat of unknown sources) and 1.2% were isolated from pet animals. The most abundant *mcr*-harboring serovars were *S.* Typhimurium (biphasic and monophasic variants) isolated from pig and pork (*n* = 159) followed by *d*-tartrate fermenting (*d*T+) *S.* Paratyphi B (formerly *S.* Java) isolated from poultry and poultry meat (*n* = 51) (Table 2). The *mcr-1* gene was found in eight different *Salmonella* serovars and was mainly associated with pig/pork and poultry/poultry meat. However, the gene was also identified in cattle/beef. The *mcr-4* gene was found in monophasic and biphasic *S.* Typhimurium, *S.* Rissen, and *S.* Ohio and was primarily associated with pigs. One *mcr-4*-harboring isolate was recovered from a quail. The *mcr-5* gene was identified in *S.* Paratyphi B *d*T+ and monophasic and biphasic *S.* Typhimurium either associated with poultry/poultry meat or pig/pork. Similarly, *mcr-9* was identified in monophasic *S.* Typhimurium from pig/pork or in *S.* Paratyphi B *d*T+ from poultry/poultry meat (Table 2 and Supplementary Table S1).

**TABLE 2:** Distribution of *mcr* genes in *Salmonella* serovars isolated from different sources.

Serovar	*mcr*-variant	Isolation source (No. of isolates/No. of isolates sequenced), [AmpC/ESBL gene identified]
Heidelberg	*mcr-1*	poultry meat (2/2), [*bla*_CMY–2_]
Infantis	*mcr-1*	pork (1) poultry meat (2)
Newport	*mcr-1*	poultry meat (1)
Ohio	*mcr-4*	pig (6/1)
Paratyphi B *d*T+	*mcr-1*	poultry (21/2) poultry meat (19/1) pork (1) pet (1) not specified meat (7) cattle (1)
	*mcr-1* and *mcr-9*	poultry (1/1) poultry meat (1/1)
	*mcr-5*	poultry (6) poultry meat (3) not specified (1)
Rissen	*mcr-4*	pig (1)
Saintpaul	*mcr-1*	poultry (3/1) poultry meat (2)
Schwarzengrund	*mcr-1*	poultry (1)
Typhimurium monophasic	*mcr-1*	pig (74/1) pork (3) pet (1) minced meat (1) not specified meat (1/1) cattle (1) beef (1) not specified (2)
	*mcr-1* and *mcr-9*	pig (5/5), [*bla*_SHV–12_] pork (1/1), [*bla*_SHV–12_]
	*mcr-4*	pig (22) pet (1) minced meat (2) not specified meat (1)
	*mcr-5*	pig (2) pork (1) not specified meat (1/1)
Typhimurium	*mcr-1*	pig (28/1) not specified (1)
	*mcr-4*	pig (19) poultry (1)
	*mcr-5*	pig (4/1)

### Antimicrobial Resistance in *mcr*-Harboring Isolates

Altogether 98.8% of the *mcr*-harboring isolates detected in the study were resistant to three or more classes of antibiotics. Phenotypical resistance to ampicillin (96.1%), sulfamethoxazole (90.9%), streptomycin (89.7%, considering only isolates tested between 2011 and 2013 as streptomycin was removed from testing plates in 2014), tetracycline (81.5%), trimethoprim (63.4%), ciprofloxacin (34.3%), nalidixic acid (29.9%), chloramphenicol (26.3%), kanamycin (13.8%), gentamicin (14.2%), and cefotaxime/ceftazidime (3.1%) was observed (Supplementary Table S1).

### Characterization of Selected Isolates Using WGS

By slide agglutination non-serotypeable *mcr*-positive isolates (*n* = 10), isolates showing phenotypic resistance to 3^rd^ generation cephalosporins (*n* = 8) or isolates harboring the newly detected *mcr-9* gene (*n* = 8) were subjected to WGS. Based on sequencing data, the serovar of all ten previously untypeable isolates could be inferred (Supplementary Tables S1, S2). Moreover, WGS data for the eight 3^rd^ generation cephalosporin-resistant isolates revealed that two *mcr-1*-positive *S.* Heidelberg strains, isolated in 2017 and 2018 from poultry meat, carried the AmpC gene *bla*_CMY–__2_ and six *mcr-1*-positive monophasic *S.* Typhimurium strains, isolated between 2013 and 2015 from pig and pork, harbored the ESBL gene *bla*_SHV–__12_. The genetic relatedness of ESBL- or AmpC-producing isolates was further investigated using a SNP-based approach. Results revealed that the six monophasic *S.* Typhimurium were closely (0-11 SNPs) and the two *S.* Heidelberg isolates were distantly (46 SNP) related to each other.

The *bla*_SHV–__12_ and *mcr-1*-harboring monophasic *S.* Typhimurium were also positive for the *mcr-9* gene in PCR. WGS data analysis revealed that these isolates harbored a *mcr-9* frameshift variant (cytosine deletion at position 1092, e.g., BioSample: SAMEA104398378). In two *S.* Paratyphi B *d*T+ isolates also tested positive for *mcr-1* and *mcr-9*, another *mcr-9* frameshift mutation (guanidine deletion at position 19, e.g., BioSample: SAMEA104396051) was observed (Supplementary Table S1).

## Discussion

We identified 254 *mcr*-harboring isolates in altogether 407 colistin-resistant *S. enterica* isolates obtained from livestock, food and pet animals collected in the frame of routine diagnostics as well as national monitoring and surveillance programs in the years 2011 to 2018 in Germany using a newly developed multiplex PCR assay detecting *mcr-6* to *mcr-9* genes in combination with a previously published *mcr-1* to *mcr-5* multiplex PCR assay (Rebelo et al., 2018). From this study, we conclude that in Germany, *mcr-1* seems to be the most common *mcr* gene found in colistin-resistant *S. enterica*, followed by *mcr-4* and *mcr-5*. These *mcr* genes have successfully emerged in at least ten *Salmonella* serovars. However, monophasic and biphasic *S.* Typhimurium from the pig production were the most prevalent *mcr*-harboring isolates, followed by *S.* Paratyphi B *dT*+ derived from poultry. This is in concordance with global observations reporting mono- and biphasic *S.* Typhimurium as the most prevalent *mcr-*positive serovars and poultry and swine as the most abundant sources of *mcr*-positive *Salmonella* isolates (Lima et al., 2019). Also other *mcr*-harboring *Salmonella* serovars including Heidelberg, Infantis, Newport, Paratyphi B *d*T+ (Java), Rissen, Saintpaul and Schwarzengrund have been previously reported. Most of them were described to harbor *mcr-1* (Lima et al., 2019). However, distribution of other *mcr* genes in *Salmonella* might be underestimated to this timepoint, as extensive screenings of strain collections for novel *mcr* genes are still ongoing. In our study, *mcr-4*- and *mcr-5*-harboring isolates were frequently identified, whereas *mcr-2*, *mcr-3*, *mcr-6*, *mcr-7*, and *mcr-8* could not be detected. The reason might be that those genes are restricted in their distribution in terms of bacterial host species or region.

In our collection, the *mcr-9* gene was found in colistin-resistant isolates also positive for *mcr-1*. WGS analysis of the respective isolates revealed that two different frameshift mutations of *mcr-9* occurred, leading probably to non-functional MCR-9 proteins. The ability of MCR-9 to cause colistin resistance was only investigated in few studies (Carroll et al., 2019; Kieffer et al., 2019). The *mcr-9* gene was initially identified in a colistin-susceptible *S.* Typhimurium. When *mcr-9* was expressed under an inducible promotor in *E. coli* NEB5α, a reduced colistin-susceptibility (MIC > 2.5 mg/L) was observed (Carroll et al., 2019). Results of a second study suggested that *mcr-9* is inducible by sub-inhibitory concentrations of colistin. Inducibility was related to a two-component regulatory system located downstream of the *mcr-9* gene (Kieffer et al., 2019). Altogether, the gene seems to be widely distributed. According to the NCBI Pathogen Detection Browser^3^, *mcr-9* is present in various Enterobacteriaceae species including *Klebsiella* spp., *E. coli*, *Enterobacter* spp. and more than 40 different *Salmonella* serovars from human and animal origin collected worldwide. Screening in our NRL *Salmonella* WGS database for additional *mcr-9* harboring isolates revealed the presence of *mcr-9.1* in eight isolates belonging to four *Salmonella* serovars (Supplementary Table S3). All these isolates were initially tested to be colistin-susceptible (MIC ≤2 mg/L) and therefore not considered for the PCR screening. Among the *mcr-9.1*-positive isolates are the carbapenem-resistant VIM-1-producing *S.* Infantis strains 15-SA01028 (BioSample: SAMN08475067) isolated from a minced meat sample and the NDM-1-producing *S.* Corvallis 12-01738 (BioSample: SAMN08685598) derived from a wild bird previously sequenced using long-read sequencing (Fischer et al., 2013; Borowiak et al., 2017b, 2018; Hadziabdic et al., 2018). Both isolates harbored the *mcr-9.1* gene on IncHI2/IncHI2A plasmids named pSE15-SA01028 and pSE12-01738-1, respectively. The two-component regulatory system associated with *mcr-9.1* inducibility is present in pSE15-SA01028 but missing in pSE12-01738-1. In our PCR study, only *S. enterica* isolates with Minimum Inhibitory Concentration (MIC) values for colistin >2 mg/L were subjected to PCR screening. However, recent studies showed that colistin MIC values determined by the recommended broth microdilution method might vary and MIC values of ≤2 mg/L can be observed, not only for *mcr-9*, but also for other *mcr*-harboring isolates (Borowiak et al., 2019; Zając et al., 2019). Thus, additional strains from the collection of the NRL *Salmonella* exposing MIC values of ≤2 mg/L might encode *mcr* genes.

On the other hand, 153 of the 407 (37.6%) initially colistin-resistant *Salmonella* isolates were not positive for known *mcr* genes. These strains could harbor chromosomal point mutations or unknown plasmid-mediated colistin resistance determinants. However, in some of the isolates, naturally decreased colistin susceptibility and fluctuations in MIC measurement might be responsible for increased MIC values (>2 mg/L).

The occurrence of *mcr* genes in monophasic and biphasic *S.* Typhimurium, *S.* Infantis and *S.* Newport have to be considered critically, as these serovars belong to top five serovars leading to human salmonellosis in Europe (EFSA, 2018). *mcr-*harboring *Salmonella* isolates can spread to humans through the consumption of undercooked food products derived from livestock. Especially worrisome is the high number of isolates resistant to three or more classes of antibiotics, which are considered as multidrug-resistant (Magiorakos et al., 2012) and the detection of ESBL- and AmpC-producing isolates among *mcr*-positive *Salmonella* strains. ESBL-producing and *mcr*-harboring *Salmonella* isolates from the animal production were already described before (Wang et al., 2017; Carfora et al., 2018). Further spread of these isolates or resistance traits limits antimicrobial treatment options for infections in man and animal and represents a major public health concern.

## Conclusion

For monitoring the spread of all known *mcr* genes in the food chain, the available *mcr-1* to *mcr-5* multiplex PCR was complemented with a novel *mcr-6* to *mcr-9* multiplex PCR assay. The protocol presented here can easily be extended to further *mcr* genes which might be detected in the future. Extensive PCR screening using a previously described and the newly developed *mcr* multiplex PCR protocol revealed that certain *mcr* genes are widely distributed among colistin-resistant *Salmonella* isolates from livestock and food in Germany. We could detect *mcr-1*, *mcr-4* and *mcr-5* genes as well as *mcr-9* frameshift variants, which have emerged in various *Salmonella* serovars derived from different livestock animals, production lines and food products. The high rate of multidrug resistance as well as the co-occurrence of ESBL and AmpC genes with *mcr-1* might threaten public health and have to be considered critically.

## Data Availability Statement

Raw sequence data can be found in the NCBI database under following BioSample accessions: SAMEA104396046, SAMEA104396051, SAMEA104396062, SAMEA104398375, SAMEA104398377, SAMEA104398378, SAMEA5192222, SAMEA5192223, SAMEA5737695, SAMEA5737696, SAMEA5737697, SAMEA5737699, SAMEA5737700, SAMEA5737701, SAMEA5737702, SAMEA5737703, SAMEA5737704, SAMEA5737705, SAMEA5737706, and SAMEA5757422.

## Author Contributions

MB and BM designed the study. JF and IS provided the samples and performed pre-analysis. MB, BB, KT, and JH performed the experiments. MB interpreted the results and wrote the draft manuscript. CD supported the bioinformatics analysis. JF, CD, JH, and BM were involved in manuscript revision prior submission of the manuscript.

## Disclaimer

The conclusion, findings, and opinions expressed in this scientific paper reflect only the view of the authors and not the official position of the European Food Safety Authority.

## Conflict of Interest

The authors declare that the research was conducted in the absence of any commercial or financial relationships that could be construed as a potential conflict of interest.

## Footnotes

1 https://enterobase.readthedocs.io/en/latest/mlst/mlst-legacy-info-senterica.html

2 https://cge.cbs.dtu.dk/services/

3 https://www.ncbi.nlm.nih.gov/pathogens/isolates#/search/AMR_genotypes:(mcr-9*)
