# Supplementary material for: Development of a Novel mcr-6 to mcr-9 Multiplex PCR and Assessment of mcr-1 to mcr-9 Occurrence in Colistin-Resistant Salmonella enterica Isolates From Environment, Feed, Animals and Food (2011–2018) in Germany
Source: Front Microbiol. 2020 Feb 4;11:80. doi: 10.3389/fmicb.2020.00080 (PMC7011100; doi:10.3389/fmicb.2020.00080)
Supplement: Supplementary file 1 [file Table_1.DOCX]

Supplementary Material

# Supplementary Table S1: Detailed information about the *mcr*-positive *Salmonella* isolates identified in this study. Isolates not serotypeable by slide agglutination, isolates resistant to 3^rd^ generation cephalosporins and isolates harboring *mcr-9* (respective traits are highlighted in bold) were sequenced for further characterization. Serovar assignment marked with “*”are based on WGS data. The *mcr-9* gene variants and ESBL/AmpC genes identified in WGS data are listed in brackets () in the BioSample column. The variant *mcr-9*-like^a^ is characterized by a cytosine deletion at position 1092 and the variant *mcr-9*-like^b^ by a guanidine deletion at position 19. Abbreviations: AMP: ampicillin, CHL: chloramphenicol, CIP: ciprofloxacin, COL: colistin, FFN: florfenicol, FOT: cefotaxime, GEN: gentamicin, KAN: kanamycin, NAL: nalidixic acid, STR: streptomycin, SMX: sulfamethoxazole, TAZ: ceftazidime, TET: tetracycline, TGC: tigecycline, TMP: trimethoprim.

| **Sample ID** | **Year** | ***Salmonella* serovar** | **Source** | **Source classification** | **Phenotypic resistance profile** | ***mcr* gene (PCR)** | **BioSample** |
| --- | --- | --- | --- | --- | --- | --- | --- |
| 11-00207-0 | 2011 | monophasic Typhimurium | pig | pig | AMP, COL, SMX, STR, TET, TMP | *mcr-4* |  |
| 11-00274-0 | 2011 | monophasic Typhimurium | pig | pig | AMP, CIP, COL, SMX, STR, TET | *mcr-4* |  |
| 11-00422-0 | 2011 | Paratyphi B *d*T+ | broiler meat | poultry meat | AMP, CIP, COL, NAL, SMX, STR, TET, TMP | *mcr-5* |  |
| 11-01025-0 | 2011 | monophasic Typhimurium | pig, feces | pig | AMP, COL, SMX, STR, TET | *mcr-1* |  |
| 11-01150-0 | 2011 | Typhimurium | pig | pig | AMP, COL, KAN, SMX, STR, TET, TMP | *mcr-1* |  |
| 11-02165-0 | 2011 | Paratyphi B *d*T+ | meat | meat | AMP, CHL, CIP, COL, GEN, KAN, NAL, SMX, STR, TET, TMP | *mcr-1* |  |
| 11-02194-0 | 2011 | Paratyphi B *d*T+ | meat | meat | AMP, CHL, CIP, NAL, SMX, STR, TET, TMP | *mcr-1* |  |
| 11-02244-0 | 2011 | monophasic Typhimurium | pig, feces | pig | AMP, COL, SMX, STR, TET | *mcr-1* |  |
| 11-02308-0 | 2011 | Typhimurium* | piglet | pig | AMP, COL, KAN, SMX, STR, TET, TMP | *mcr-1* | SAMEA5737697 |
| 11-02337-0 | 2011 | Typhimurium | piglet, feces | pig | AMP, COL, SMX, STR, TET, TMP | *mcr-4* |  |
| 11-02585-0 | 2011 | Ohio* | pig | pig | AMP, COL | *mcr-4* | SAMEA5737699 |
| 11-02674-0 | 2011 | monophasic Typhimurium | pig, feces | pig | AMP, COL, SMX, STR, TET | *mcr-1* |  |
| 11-02845-0 | 2011 | Typhimurium | piglet, feces | pig | AMP, COL, SMX, STR, TET, TMP | *mcr-4* |  |
| 11-03155-0 | 2011 | Typhimurium | pig, feces | pig | AMP, COL, KAN, SMX, STR, TET, TMP | *mcr-5* |  |
| 11-03506-0 | 2011 | Typhimurium | piglet | pig | AMP, COL, SMX, STR, TET, TMP | *mcr-1* |  |
| 11-03654-0 | 2011 | Paratyphi B *d*T+ | poultry | poultry | AMP, CHL, CIP, COL, FFN, NAL, SMX, STR, TET, TMP | *mcr-1* |  |
| 11-03671-0 | 2011 | monophasic Typhimurium | pig, feces | pig | AMP, COL, SMX, STR, TET | *mcr-5* |  |
| 11-03770-0 | 2011 | Paratyphi B *d*T+ | broiler meat | poultry meat | AMP, CHL, CIP, COL, NAL, SMX, STR, TET, TMP | *mcr-1* |  |
| 11-04048-0 | 2011 | monophasic Typhimurium | pig, feces | pig | AMP, COL, SMX, STR, TET | *mcr-1* |  |
| 11-04054-0 | 2011 | Paratyphi B *d*T+ | broiler, skin | poultry | AMP, CIP, COL, GEN, KAN, NAL, SMX, STR, TET, TMP | *mcr-1* |  |
| 11-04067-0 | 2011 | monophasic Typhimurium | piglet | pig | AMP, COL, TET | *mcr-4* |  |
| 11-04292-0 | 2011 | Typhimurium | pig, feces | pig | AMP, COL, SMX, STR, TET, TMP | *mcr-4* |  |
| 11-04511-0 | 2011 | Typhimurium | piglet, feces | pig | AMP, COL, SMX, STR, TET, TMP | *mcr-4* |  |
| 11-04559-0 | 2011 | Paratyphi B *d*T+ | broiler meat | poultry meat | AMP, CHL, CIP, COL, NAL, SMX, STR, TET, TMP | *mcr-1* |  |
| 12-00210-0 | 2012 | Paratyphi B *d*T+ | dog | pet | AMP, CHL, CIP, COL, NAL, SMX, STR, TET, TMP | *mcr-1* |  |
| 12-00300-0 | 2012 | Paratyphi B *d*T+ | broiler meat | poultry meat | AMP, CIP, COL, NAL, SMX, TMP | *mcr-1* |  |
| 12-00331-0 | 2012 | Typhimurium | piglet | pig | AMP, COL, SMX, STR, TET, TMP | *mcr-1* |  |
| 12-00337-0 | 2012 | Typhimurium | pig | pig | AMP, COL, SMX, STR, TET, TMP | *mcr-1* |  |
| 12-00369-0 | 2012 | Typhimurium | pig, feces | pig | AMP, COL, SMX, STR, TET, TMP | *mcr-4* |  |
| 12-00413-0 | 2012 | Saintpaul | turkey meat | poultry meat | AMP, CIP, COL, GEN, KAN, NAL, SMX, STR | *mcr-1* |  |
| 12-00414-0 | 2012 | Saintpaul* | turkey | poultry | AMP, CIP, COL, GEN, KAN, NAL, SMX, STR | *mcr-1* | SAMEA5737700 |
| 12-00555-0 | 2012 | Paratyphi B *d*T+ | broiler meat | poultry meat | AMP, CHL, CIP, COL, FFN, NAL, SMX, STR, TET, TMP | *mcr-1* |  |
| 12-00591-0 | 2012 | monophasic Typhimurium | pig, feces | pig | AMP, COL, SMX, STR, TET | *mcr-1* |  |
| 12-00614-0 | 2012 | Paratyphi B *d*T+ | broiler meat | poultry meat | AMP, CHL, CIP, COL, NAL, SMX, STR, TET, TMP | *mcr-1* |  |
| 12-00618-0 | 2012 | Paratyphi B *d*T+ | poultry | poultry | CIP, COL, NAL, TMP | *mcr-5* |  |
| 12-00619-0 | 2012 | Paratyphi B *d*T+ | poultry | poultry | AMP, CIP, COL, NAL, SMX, STR, TET, TMP | *mcr-5* |  |
| 12-00663-1 | 2012 | Paratyphi B *d*T+ | poultry | poultry | AMP, CIP, COL, NAL, SMX, STR, TET, TMP | *mcr-5* |  |
| 12-00743-0 | 2012 | Paratyphi B *d*T+ | broiler meat | poultry meat | AMP, CHL, CIP, COL, NAL, SMX, STR, TET, TMP | *mcr-1* |  |
| 12-00956-0 | 2012 | monophasic Typhimurium | pig, feces | pig | AMP, COL, SMX, STR, TET | *mcr-1* |  |
| 12-00966-0 | 2012 | monophasic Typhimurium | pig | pig | AMP, CHL, COL, FFN, SMX, STR, TET | *mcr-1* |  |
| 12-01000-0 | 2012 | monophasic Typhimurium | pig, feces | pig | AMP, COL, SMX, STR, TET | *mcr-1* |  |
| 12-01063-0 | 2012 | Paratyphi B *d*T+ | broiler meat | poultry meat | AMP, CIP, COL, NAL, SMX, STR, TMP | *mcr-1* |  |
| 12-01153-0 | 2012 | Typhimurium | unknown | unknown | AMP, COL, KAN, SMX, STR, TET, TMP | *mcr-1* |  |
| 12-01200-0 | 2012 | Typhimurium | piglet, feces | pig | AMP, COL, SMX, STR, TET, TMP | *mcr-4* |  |
| 12-01208-0 | 2012 | Paratyphi B *d*T+ | broiler meat | poultry meat | AMP, CHL, CIP, COL, NAL, SMX, STR, TET, TMP | *mcr-1* |  |
| 12-01212-0 | 2012 | Paratyphi B *d*T+ | broiler meat | poultry meat | AMP, CHL, CIP, COL, GEN, KAN, NAL, SMX, STR, TET, TMP | *mcr-1* |  |
| 12-01240-0 | 2012 | Typhimurium | piglet, feces | pig | AMP, CHL, CIP, COL, FFN, NAL, SMX, STR, TET | *mcr-4* |  |
| 12-01256-0 | 2012 | Saintpaul | poultry | poultry | AMP, CIP, COL, GEN, KAN, NAL, SMX, STR | *mcr-1* |  |
| 12-01405-0 | 2012 | Saintpaul | turkey meat | poultry meat | AMP, CIP, COL, KAN, NAL, STR | *mcr-1* |  |
| 12-01438-0 | 2012 | Paratyphi B *d*T+ | broiler meat | poultry meat | AMP, CHL, CIP, COL, FFN, NAL, SMX, STR, TET, TMP | *mcr-1* |  |
| 12-01497-0 | 2012 | Paratyphi B *d*T+ | poultry | poultry | AMP, CIP, COL, NAL, SMX, TET, TMP | *mcr-5* |  |
| 12-01512-0 | 2012 | Paratyphi B *d*T+* | broiler meat | poultry meat | AMP, CHL, CIP, COL, NAL, SMX, STR, TET, TMP | *mcr-1* | SAMEA5737701 |
| 12-01549-0 | 2012 | Paratyphi B *d*T+ | turkey, sock swab | poultry | AMP, CIP, COL, NAL, SMX, STR, TET, TMP | *mcr-5* |  |
| 12-01690-0 | 2012 | Typhimurium | pig, feces | pig | AMP, COL, KAN, SMX, STR, TET, TMP | *mcr-1* |  |
| 12-01744-0 | 2012 | Typhimurium | pig | pig | AMP, COL, KAN, SMX, STR, TET, TMP | *mcr-1* |  |
| 12-01746-0 | 2012 | monophasic Typhimurium | piglet, organs | pig | AMP, COL, TET | *mcr-4* |  |
| 12-01836-0 | 2012 | monophasic Typhimurium | pig, feces | pig | AMP, COL, KAN, SMX, STR, TET, TMP | *mcr-1* |  |
| 12-01840-0 | 2012 | monophasic Typhimurium | pig, feces | pig | AMP, CHL, COL, GEN, KAN, SMX, STR, TET, TMP | *mcr-1* |  |
| 12-02006-0 | 2012 | Ohio | pig | pig | AMP, CHL, CIP, COL, FFN, KAN, SMX, STR, TET, TMP | *mcr-4* |  |
| 12-02061-0 | 2012 | monophasic Typhimurium | calf | cow | AMP, CHL, COL, GEN, KAN, SMX, STR, TET, TMP | *mcr-1* |  |
| 12-02065-0 | 2012 | monophasic Typhimurium | pig, feces | pig | AMP, COL, SMX, STR, TET | *mcr-1* |  |
| 12-02147-0 | 2012 | Typhimurium | piglet | pig | AMP, COL, SMX, STR, TET, TMP | *mcr-4* |  |
| 12-02148-0 | 2012 | Typhimurium | piglet | pig | AMP, COL, SMX, STR, TET, TMP | *mcr-4* |  |
| 12-02227-0 | 2012 | Typhimurium | piglet, feces | pig | AMP, COL, SMX, STR, TET, TMP | *mcr-4* |  |
| 12-02228-0 | 2012 | Typhimurium | piglet, feces | pig | AMP, COL, SMX, STR, TET, TMP | *mcr-4* |  |
| 12-02284-0 | 2012 | monophasic Typhimurium | pork | pork | AMP, COL, KAN, SMX, STR, TET, TMP | *mcr-5* |  |
| 12-02447-0 | 2012 | monophasic Typhimurium | pig, feces | pig | AMP, COL, SMX, STR, TET | *mcr-1* |  |
| 12-02539-0 | 2012 | Paratyphi B *d*T+ | unknown | unknown | CIP, COL, NAL, TMP | *mcr-5* |  |
| 12-02544-0 | 2012 | Paratyphi B *d*T+ | poultry | poultry | AMP, CIP, COL, NAL, SMX, TET, TMP | *mcr-5* |  |
| 12-02653-0 | 2012 | Saintpaul | poultry | poultry | AMP, CIP, COL, GEN, NAL, SMX, STR | *mcr-1* |  |
| 12-02784-0 | 2012 | Typhimurium | pig | pig | AMP, COL, KAN, SMX, STR, TET, TMP | *mcr-5* |  |
| 12-02811-0 | 2012 | Paratyphi B *d*T+ | cattle, organs | cow | CIP, COL, NAL, SMX, STR, TMP | *mcr-1* |  |
| 12-02926-0 | 2012 | Ohio | pig | pig | AMP, COL, GEN, SMX, STR, TET, TMP | *mcr-4* |  |
| 12-02927-0 | 2012 | monophasic Typhimurium | pig | pig | AMP, COL, SMX, STR, TET | *mcr-4* |  |
| 12-03294-0 | 2012 | Paratyphi B *d*T+ | meat | meat | AMP, CIP, COL, NAL, SMX, STR, TMP | *mcr-1* |  |
| 12-03478-0 | 2012 | monophasic Typhimurium | piglet, feces | pig | AMP, CIP, COL, SMX, STR, TET | *mcr-4* |  |
| 12-03489-0 | 2012 | monophasic Typhimurium | piglet | pig | AMP, CHL, COL, GEN, KAN, SMX, STR, TET, TMP | *mcr-1* |  |
| 12-03724-0 | 2012 | Paratyphi B *d*T+ | broiler meat | poultry meat | AMP, CIP, COL, NAL, SMX, STR, TET, TMP | *mcr-5* |  |
| 12-03814-0 | 2012 | Typhimurium | piglet | pig | AMP, COL, KAN, SMX, STR, TET, TMP | *mcr-1* |  |
| 12-03826-0 | 2012 | Typhimurium | piglet | pig | AMP, COL, SMX, STR, TET, TMP | *mcr-1* |  |
| 12-03831-0 | 2012 | monophasic Typhimurium | piglet | pig | AMP, COL, TET | *mcr-4* |  |
| 12-SA03843-0 | 2012 | monophasic Typhimurium | pig | pig | AMP, CHL, COL, GEN, KAN, SMX, STR, TET, TMP | *mcr-1* |  |
| 12-SA03903-0 | 2012 | Paratyphi B *d*T+ | broiler meat | poultry meat | AMP, CIP, COL, NAL, SMX, STR, TMP | *mcr-1* |  |
| 12-SA04048-0 | 2012 | Typhimurium | pig | pig | AMP, COL, SMX, STR, TET, TMP | *mcr-4* |  |
| 12-SA04093-0 | 2012 | monophasic Typhimurium | pig, feces | pig | AMP, CHL, COL, GEN, KAN, SMX, STR, TET, TMP | *mcr-1* |  |
| 12-SA04122-0 | 2012 | Paratyphi B *d*T+ | poultry | poultry | AMP, CIP, COL, NAL, SMX, STR, TMP | *mcr-1* |  |
| 12-SA04157-0 | 2012 | Typhimurium | pig, feces | pig | AMP, COL, SMX, STR, TET, TMP | *mcr-5* |  |
| 12-SA04270-0 | 2012 | monophasic Typhimurium | pig | pig | AMP, CHL, COL, FFN, SMX, STR, TET, TMP | *mcr-4* |  |
| 13-SA00018-0 | 2013 | monophasic Typhimurium | pork | pork | AMP, CIP, COL, **FOT**, GEN, KAN, SMX, STR, **TAZ**, TET, TMP | *mcr-1,* ***mcr-9*** | SAMEA104398375 (*mcr-9*-like^a^,  *bla*_SHV-12_^)^ |
| 13-SA00260-0 | 2013 | Rissen | piglet | pig | AMP, CHL, COL, GEN, SMX, STR, TET, TMP | *mcr-4* |  |
| 13-SA00276-0 | 2013 | Ohio | pig, organs | pig | AMP, CHL, CIP, COL, FFN, KAN, SMX, STR, TET, TMP | *mcr-4* |  |
| 13-SA00281-0 | 2013 | Typhimurium | pig | pig | AMP, COL, KAN, SMX, STR, TET, TMP | *mcr-4* |  |
| 13-SA00395-0 | 2013 | Paratyphi B *d*T+ | broiler, feces | poultry | AMP, CIP, COL, NAL, SMX, STR, TMP | *mcr-1* |  |
| 13-SA00569-0 | 2013 | Typhimurium* | pig, feces | pig | AMP, COL, SMX, STR, TET, TMP | *mcr-5* | SAMEA5192222 |
| 13-SA00617-0 | 2013 | Paratyphi B *d*T+ | broiler, organs | poultry meat | AMP, CIP, COL, NAL, SMX, STR, TMP | *mcr-1* |  |
| 13-SA01136-0 | 2013 | monophasic Typhimurium* | meat | meat | AMP, COL, SMX, STR, TET | *mcr-1* | SAMEA5737702 |
| 13-SA01257-0 | 2013 | monophasic Typhimurium | sausage | meat | AMP, COL, SMX, STR, TET | *mcr-4* |  |
| 13-SA01391-0 | 2013 | monophasic Typhimurium | pig | pig | AMP, COL, SMX, STR, TET | *mcr-1* |  |
| 13-SA01718-0 | 2013 | Paratyphi B *d*T+ | broiler meat | poultry meat | AMP, CIP, COL, NAL, SMX, STR, TET, TMP | *mcr-5* |  |
| 13-SA01735-0 | 2013 | monophasic Typhimurium | pig | pig | AMP, CHL, COL, GEN, KAN, SMX, STR, TET, TMP | *mcr-1* |  |
| 13-SA01881-0 | 2013 | monophasic Typhimurium | unknown | unknown | AMP, CHL, COL, GEN, KAN, SMX, STR, TET, TMP | *mcr-1* |  |
| 13-SA01890-0 | 2013 | monophasic Typhimurium | pig, feces | pig | AMP, CHL, COL, GEN, KAN, SMX, STR, TET, TMP | *mcr-1* |  |
| 13-SA01943-0 | 2013 | monophasic Typhimurium | pig | pig | AMP, CHL, COL, GEN, KAN, SMX, STR, TET, TMP | *mcr-1* |  |
| 13-SA02194-0 | 2013 | Paratyphi B *d*T+ | poultry | poultry | AMP, CIP, COL, NAL, SMX, TMP | *mcr-1* |  |
| 13-SA02281-0 | 2013 | Paratyphi B *d*T+ | broiler, skin | poultry | AMP, CHL, CIP, COL, NAL, SMX, STR, TET, TMP | *mcr-1* |  |
| 13-SA02283-0 | 2013 | Paratyphi B *d*T+ | broiler meat | poultry meat | AMP, CIP, COL, NAL, STR, TMP | *mcr-1* |  |
| 13-SA02299-0 | 2013 | Paratyphi B *d*T+ | meat | meat | AMP, CIP, COL, NAL, SMX, STR, TMP | *mcr-1* |  |
| 13-SA02434-0 | 2013 | Paratyphi B *d*T+ | broiler, sock swab | poultry | AMP, CIP, COL, NAL, SMX, TMP | *mcr-1* |  |
| 13-SA02497-0 | 2013 | monophasic Typhimurium | pig, feces | pig | AMP, CHL, COL, GEN, KAN, SMX, STR, TET, TMP | *mcr-1* |  |
| 13-SA02530-0 | 2013 | monophasic Typhimurium | pig, organs | pig | AMP, CHL, COL, SMX, TET | *mcr-4* |  |
| 13-SA02656-0 | 2013 | Paratyphi B *d*T+ | broiler meat | poultry meat | CIP, COL, NAL, STR, TMP | *mcr-1,* ***mcr-9*** | SAMEA104396046 (*mcr-9*-like^b^) |
| 13-SA02717-0 | 2013 | monophasic Typhimurium* | meat | meat | AMP, COL, SMX, STR, TMP | *mcr-5* | SAMEA5192223 |
| 13-SA02788-0 | 2013 | Paratyphi B *d*T+ | broiler meat | poultry meat | AMP, CIP, COL, NAL, STR, TMP | *mcr-1* |  |
| 13-SA02803-0 | 2013 | Paratyphi B *d*T+ | broiler meat | poultry meat | AMP, CIP, COL, NAL, SMX, STR, TMP | *mcr-1* |  |
| 13-SA03134-0 | 2013 | Typhimurium | pig | pig | AMP, COL, KAN, SMX, STR, TET, TMP | *mcr-4* |  |
| 13-SA03212-0 | 2013 | monophasic Typhimurium | pig | pig | AMP, CHL, COL, SMX, STR, TET, TMP | *mcr-1* |  |
| 14-SA00036-0 | 2014 | Typhimurium | piglet | pig | AMP, COL, KAN, SMX, STR, TET, TMP | *mcr-4* |  |
| 14-SA00052-0 | 2014 | monophasic Typhimurium | dog, feces | pet | COL, TET | *mcr-4* |  |
| 14-SA00169-0 | 2014 | monophasic Typhimurium | piglet | pig | AMP, CIP, COL, **FOT**, GEN, KAN, SMX, STR, **TAZ**, TET, TMP | *mcr-1,* ***mcr-9*** | SAMEA104398377 (*mcr-9*-like^a^,  *bla*_SHV-12_^)^ |
| 14-SA00170-0 | 2014 | monophasic Typhimurium | piglet | pig | AMP, COL, **FOT**, GEN, KAN, SMX, STR, **TAZ**, TET, TMP | *mcr-1,* ***mcr-9*** | SAMEA104398378 (*mcr-9*-like^a^,  *bla*_SHV-12_^)^ |
| 14-SA00221-0 | 2014 | monophasic Typhimurium | pig, liver | pork | AMP, CIP, COL, SMX, STR, TET | *mcr-1* |  |
| 14-SA00268-0 | 2014 | Schwarzengrund | broiler | poultry | CIP, COL | *mcr-1* |  |
| 14-SA00333-0 | 2014 | Paratyphi B *d*Ta+ | broiler, skin | poultry | AMP, CHL, CIP, COL, NAL, SMX, STR, TET, TMP | *mcr-1* |  |
| 14-SA00360-0 | 2014 | monophasic Typhimurium | pig | pig | AMP, COL, SMX, STR, TET | *mcr-4* |  |
| 14-SA00775-0 | 2014 | Paratyphi B *d*Ta+ | broiler, skin | poultry | AMP, CIP, COL, NAL, TMP | *mcr-1* |  |
| 14-SA00777-0 | 2014 | Paratyphi B *d*Ta+ | broiler, skin | poultry | AMP, CHL, CIP, COL, NAL, SMX, TMP | *mcr-1* |  |
| 14-SA00918-0 | 2014 | Paratyphi B *d*Ta+ | broiler, skin | poultry | CIP, COL, NAL, TMP | *mcr-1,* ***mcr-9*** | SAMEA104396051 (*mcr-9*-like^b^) |
| 14-SA01149-0 | 2014 | Paratyphi B *d*Ta+ | meat | meat | AMP, CHL, CIP, COL, NAL, SMX, TET, TGC, TMP | *mcr-1* |  |
| 14-SA02221-0 | 2014 | monophasic Typhimurium | piglet | pig | AMP, COL, SMX, TET, TMP | *mcr-1* |  |
| 14-SA02325-0 | 2014 | monophasic Typhimurium | piglet | pig | AMP, COL, **FOT**, GEN, SMX, **TAZ**, TET, TMP | *mcr-1 ,* ***mcr-9*** | SAMEA5757422 (*mcr-9*-like^a^,  *bla*_SHV-12_^)^ |
| 14-SA02330-0 | 2014 | Typhimurium | piglet | pig | AMP, COL, SMX, TET, TMP | *mcr-4* |  |
| 14-SA02381-0 | 2014 | monophasic Typhimurium | pig | pig | AMP, COL, SMX, TET | *mcr-4* |  |
| 14-SA02404-0 | 2014 | Typhimurium | piglet | pig | AMP, COL, SMX, TET, TMP | *mcr-1* |  |
| 14-SA02489-0 | 2014 | monophasic Typhimurium | pig | pig | AMP, COL, SMX, TET | *mcr-1* |  |
| 14-SA02536-0 | 2014 | Paratyphi B *d*T+ | meat | meat | AMP, CHL, CIP, COL, NAL, SMX, TET, TMP | *mcr-1* |  |
| 14-SA02575-0 | 2014 | Typhimurium | pig | pig | AMP, CHL, COL, SMX, TET, TMP | *mcr-1* |  |
| 14-SA02597-0 | 2014 | Typhimurium | pig | pig | AMP, COL, SMX, TET | *mcr-1* |  |
| 14-SA02723-0 | 2014 | monophasic Typhimurium | pig, feces | pig | AMP, COL, SMX, TET | *mcr-1* |  |
| 14-SA02725-0 | 2014 | monophasic Typhimurium | piglet, feces | pig | AMP, COL, SMX | *mcr-1* |  |
| 14-SA02741-0 | 2014 | Paratyphi B *d*T+ | meat | meat | AMP, CHL, CIP, COL, NAL, SMX, TET, TMP | *mcr-1* |  |
| 14-SA02795-0 | 2014 | monophasic Typhimurium | piglet, organs | pig | AMP, CHL, COL, SMX, TET, TMP | *mcr-1* |  |
| 14-SA02806-0 | 2014 | Typhimurium | pig | pig | AMP, COL, SMX, TET, TMP | *mcr-1* |  |
| 14-SA02860-0 | 2014 | Paratyphi B *d*T+ | broiler meat | poultry meat | AMP, CHL, CIP, COL, NAL, SMX, TET, TMP | *mcr-1* |  |
| 14-SA02865-0 | 2014 | monophasic Typhimurium | pig | pig | AMP, CIP, COL, SMX, TET, TMP | *mcr-1* |  |
| 14-SA02867-0 | 2014 | monophasic Typhimurium | pig | pig | AMP, COL, SMX, TET, TMP | *mcr-1* |  |
| 14-SA02874-0 | 2014 | monophasic Typhimurium | pig | pig | AMP, CHL, COL, SMX, TET | *mcr-1* |  |
| 14-SA02875-0 | 2014 | monophasic Typhimurium | pig | pig | AMP, COL, SMX, TET, TMP | *mcr-1* |  |
| 14-SA03087-0 | 2014 | Typhimurium | pig | pig | AMP, CHL, COL, SMX, TET, TMP | *mcr-1* |  |
| 14-SA03215-0 | 2014 | monophasic Typhimurium | pig | pig | AMP, COL, SMX, TET | *mcr-1* |  |
| 15-SA00146-0 | 2015 | Paratyphi B *d*T+ | broiler meat | poultry meat | AMP, CIP, COL, NAL, TMP | *mcr-1* |  |
| 15-SA00147-0 | 2015 | Paratyphi B *d*T+ | broiler meat | poultry meat | AMP, CIP, COL, NAL, TMP | *mcr-1* |  |
| 15-SA00386-0 | 2015 | monophasic Typhimurium | pig | pig | AMP, COL, SMX, TET | *mcr-1* |  |
| 15-SA00407-0 | 2015 | monophasic Typhimurium | pig | pig | AMP, CHL, COL, TET, TMP | *mcr-1* |  |
| 15-SA00591-0 | 2015 | Infantis | broiler, liver | poultry meat | CIP, COL, NAL, SMX, TET | *mcr-1* |  |
| 15-SA00614-0 | 2015 | monophasic Typhimurium | pig, feces | pig | AMP, COL, SMX, TET | *mcr-1* |  |
| 15-SA00639-0 | 2015 | monophasic Typhimurium | pig | pig | AMP, COL, SMX, TET | *mcr-1* |  |
| 15-SA00756-0 | 2015 | monophasic Typhimurium | pig, feces | pig | AMP, COL, SMX, TET | *mcr-1* |  |
| 15-SA00820-0 | 2015 | Typhimurium | quail | poultry | AMP, CHL, COL, TET | *mcr-4* |  |
| 15-SA00894-0 | 2015 | monophasic Typhimurium | pig, organs | pig | AMP, COL, SMX, TET | *mcr-1* |  |
| 15-SA00963-0 | 2015 | monophasic Typhimurium | minced meat (pork/beef) | minced meat | AMP, COL, SMX, TET | *mcr-4* |  |
| 15-SA01188-0 | 2015 | Typhimurium | pig, organs | pig | AMP, COL, SMX, TET, TMP | *mcr-1* |  |
| 15-SA01265-0 | 2015 | monophasic Typhimurium | pig, sock swabs | pig | AMP, COL, SMX, TET | *mcr-1* |  |
| 15-SA01381-0 | 2015 | monophasic Typhimurium | piglet, organs | pig | AMP, COL, **FOT**, GEN, SMX, **TAZ**, TET, TMP | *mcr-1,* ***mcr-9*** | SAMEA5737695  (*mcr-9*-like^a^,  *bla*_SHV-12_^)^ |
| 15-SA01424-0 | 2015 | Typhimurium | pig, kidney | pig | AMP, COL, SMX, TET, TMP | *mcr-4* |  |
| 15-SA01523-0 | 2015 | Paratyphi B *d*T+ | pork | pork | AMP, CIP, COL, NAL, SMX, TMP | *mcr-1* |  |
| 15-SA01528-0 | 2015 | monophasic Typhimurium | pork tongue | pork | AMP, CHL, CIP, COL, GEN, SMX, TET, TMP | *mcr-1* |  |
| 15-SA01602-0 | 2015 | monophasic Typhimurium | pig, feces | pig | AMP, COL, SMX, TET | *mcr-1* |  |
| 15-SA01681-0 | 2015 | Typhimurium | pig | pig | AMP, CHL, COL, SMX, TET, TMP | *mcr-1* |  |
| 15-SA01864-0 | 2015 | monophasic Typhimurium | piglet | pig | AMP, COL, **FOT**, GEN, SMX, **TAZ**, TET, TMP | *mcr-1,* ***mcr-9*** | SAMEA5737696 (*mcr-9*-like^a^,  *bla*_SHV-12_^)^ |
| 15-SA01949-0 | 2015 | Paratyphi B *d*T+ | broiler fat tissue | poultry | AMP, CHL, CIP, COL, NAL, SMX, TET, TMP | *mcr-1* |  |
| 15-SA01997-0 | 2015 | Newport | turkey meat | poultry meat | AMP, CIP, COL, NAL, TET | *mcr-1* |  |
| 15-SA02035-0 | 2015 | monophasic Typhimurium | piglet | pig | AMP, COL, SMX, TET | *mcr-1* |  |
| 15-SA02189-0 | 2015 | monophasic Typhimurium | piglet | pig | AMP, COL, SMX, TET | *mcr-1* |  |
| 15-SA02221-0 | 2015 | monophasic Typhimurium | pig | pig | AMP, CHL, COL, SMX, TET | *mcr-1* |  |
| 15-SA02338-0 | 2015 | Paratyphi B *d*T+ | broiler, heart | poultry | AMP, CIP, COL, NAL, SMX, TMP | *mcr-1* |  |
| 15-SA02393-0 | 2015 | monophasic Typhimurium | pig | pig | AMP, CHL, COL, GEN, SMX, TET, TMP | *mcr-1* |  |
| 15-SA02522-0 | 2015 | monophasic Typhimurium | pig, organs | pig | AMP, COL, SMX, TET | *mcr-1* |  |
| 15-SA02571-0 | 2015 | Typhimurium | piglet | pig | AMP, COL, SMX, TET, TMP | *mcr-1* |  |
| 15-SA02653-0 | 2015 | Paratyphi B *d*T+ | broiler, organs | poultry | AMP, CIP, COL, NAL, SMX, TMP | *mcr-1* |  |
| 15-SA02671-0 | 2015 | monophasic Typhimurium | pig | pig | AMP, COL, SMX, TET | *mcr-4* |  |
| 15-SA02829-0 | 2015 | Paratyphi B *d*T+* | broiler | poultry | AMP, CIP, COL, NAL, SMX, TMP | *mcr-1* | SAMEA104396062 |
| 15-SA02945-0 | 2015 | monophasic Typhimurium | minced meat | minced meat | AMP, COL, SMX, TET | *mcr-4* |  |
| 16-SA00158-0 | 2016 | monophasic Typhimurium | pig | pig | AMP, CHL, COL, SMX | *mcr-1* |  |
| 16-SA00159-0 | 2016 | monophasic Typhimurium | pig | pig | AMP, CHL, COL, SMX, TET | *mcr-1* |  |
| 16-SA00165-0 | 2016 | monophasic Typhimurium | pig | pig | AMP, COL, SMX, TET | *mcr-1* |  |
| 16-SA00482-0 | 2016 | monophasic Typhimurium | piglet | pig | AMP, CHL, COL, GEN, SMX, TET, TMP | *mcr-1* |  |
| 16-SA00487-0 | 2016 | monophasic Typhimurium | pig | pig | AMP, COL, SMX, TET | *mcr-4* |  |
| 16-SA01009-0 | 2016 | monophasic Typhimurium | pig, feces | pig | AMP, CHL, COL, GEN, SMX, TMP | *mcr-4* |  |
| 16-SA01244-0 | 2016 | Paratyphi B *d*T+ | broiler, skin | poultry | AMP, CIP, COL, NAL, SMX, TMP | *mcr-1* |  |
| 16-SA01337-0 | 2016 | Paratyphi B *d*T+ | turkey, skin | poultry | AMP, CIP, COL, NAL, SMX, TMP | *mcr-1* |  |
| 16-SA01428-0 | 2016 | monophasic Typhimurium | pig, sock swabs | pig | AMP, COL, SMX, TET | *mcr-4* |  |
| 16-SA01429-0 | 2016 | monophasic Typhimurium | pig, sock swabs | pig | AMP, COL, SMX, TET | *mcr-4* |  |
| 16-SA01557-0 | 2016 | Typhimurium | pig, organs | pig | AMP, COL, SMX, TET, TGC, TMP | *mcr-1* |  |
| 16-SA01597-0 | 2016 | monophasic Typhimurium | pig | pig | AMP, COL, TET | *mcr-1* |  |
| 16-SA01769-0 | 2016 | monophasic Typhimurium | pig, sock swabs | pig | AMP, COL, SMX, TET, TMP | *mcr-1* |  |
| 16-SA01987-0 | 2016 | Typhimurium | pig, organs | pig | AMP, COL, SMX, TET, TMP | *mcr-1* |  |
| 16-SA02404-0 | 2016 | monophasic Typhimurium | pig, feces | pig | AMP, CHL, COL, SMX, TET, TMP | *mcr-5* |  |
| 16-SA02534-0 | 2016 | Ohio | piglet, organs | pig | AMP, COL, GEN, SMX, TET, TMP | *mcr-4* |  |
| 16-SA02629-0 | 2016 | monophasic Typhimurium | pig, feces | pig | AMP, COL, SMX | *mcr-4* |  |
| 16-SA02720-0 | 2016 | Typhimurium | pig, feces | pig | AMP, COL, SMX, TET, TMP | *mcr-1* |  |
| 16-SA02808-2 | 2016 | Paratyphi B *d*T+* | broiler | poultry | AMP, CIP, COL, NAL, SMX, TMP | *mcr-1* | SAMEA5737703 |
| 16-SA02829-0 | 2016 | monophasic Typhimurium | pig, organs | pig | AMP, COL, SMX, TET | *mcr-4* |  |
| 16-SA02832-0 | 2016 | Paratyphi B *d*T+ | broiler, cecum | poultry | AMP, CIP, COL, NAL, SMX, TMP | *mcr-1* |  |
| 16-SA02843-0 | 2016 | Paratyphi B *d*T+ | broiler, skin | poultry | AMP, CIP, COL, NAL, TMP | *mcr-1* |  |
| 16-SA02861-0 | 2016 | Paratyphi B *d*T+ | broiler, skin | poultry | AMP, CIP, COL, NAL, SMX, TMP | *mcr-1* |  |
| 16-SA03081-0 | 2016 | monophasic Typhimurium | pig, sock swabs | pig | AMP, CHL, COL, SMX, TMP | *mcr-4* |  |
| 16-SA03181-0 | 2016 | monophasic Typhimurium | unknown | unknown | AMP, COL, SMX, TET | *mcr-1* |  |
| 16-SA03297-0 | 2016 | monophasic Typhimurium | pig, organs | pig | AMP, COL, SMX, TET | *mcr-4* |  |
| 16-SA03302-0 | 2016 | monophasic Typhimurium | pig, organs | pig | AMP, COL, SMX, TET | *mcr-4* |  |
| 17-SA00266-0 | 2017 | monophasic Typhimurium | pig, gut | pig | AMP, COL, SMX, TET | *mcr-4* |  |
| 17-SA00267-0 | 2017 | Ohio | pig, abrasive sponge sample | pig | AMP, COL, GEN, SMX, TET, TMP | *mcr-4* |  |
| 17-SA00430-0 | 2017 | monophasic Typhimurium | pig | pig | AMP, COL, SMX, TET, TMP | *mcr-1* |  |
| 17-SA00624-0 | 2017 | monophasic Typhimurium | pig, abrasive sponge sample | pig | AMP, COL, SMX, TET | *mcr-1* |  |
| 17-SA01133-0 | 2017 | Infantis | sausage | pork | CIP, COL, NAL, SMX, TET | *mcr-1* |  |
| 17-SA01134-0 | 2017 | Infantis | poultry meat | poultry meat | CIP, COL, NAL, SMX, TET | *mcr-1* |  |
| 17-SA01262-0 | 2017 | Typhimurium | pig, cecum | pig | AMP, CHL, COL, SMX, TET | *mcr-1* |  |
| 17-SA01306-0 | 2017 | Paratyphi B *d*T+ | broiler | poultry | AMP, CIP, COL, NAL, SMX, TMP | *mcr-1* |  |
| 17-SA01366-0 | 2017 | monophasic Typhimurium | pig | pig | AMP, CHL, CIP, COL, SMX, TET, TGC, TMP | *mcr-1* |  |
| 17-SA01380-0 | 2017 | monophasic Typhimurium | piglet, feces | pig | AMP, COL, SMX, TET | *mcr-1* |  |
| 17-SA01385-0 | 2017 | monophasic Typhimurium* | pig, feces | pig | AMP, CHL, COL, SMX, TET, TMP | *mcr-1* | SAMEA5737704 |
| 17-SA01527-0 | 2017 | monophasic Typhimurium | pig | pig | AMP, COL, SMX, TET, TMP | *mcr-1* |  |
| 17-SA01686-0 | 2017 | monophasic Typhimurium | pig, organs | pig | AMP, CHL, COL, GEN, SMX, TET, TMP | *mcr-1* |  |
| 17-SA02115-0 | 2017 | monophasic Typhimurium | pig, feces | pig | AMP, COL, SMX, TET | *mcr-1* |  |
| 17-SA02191-0 | 2017 | monophasic Typhimurium | minced meat | minced meat | AMP, CHL, CIP, COL, GEN, NAL, SMX, TET, TMP | *mcr-1* |  |
| 17-SA02205-0 | 2017 | Typhimurium | pig, feces | pig | AMP, CHL, COL, SMX, TET, TGC | *mcr-1* |  |
| 17-SA02555-0 | 2017 | Heidelberg | broiler meat | poultry meat | AMP, CIP, COL, **FOT**, NAL, SMX, **TAZ**, TET | *mcr-1* | SAMEA5737705 (*bla*_CMY-2_) |
| 17-SA02597-0 | 2017 | monophasic Typhimurium | piglet, organs | pig | AMP, COL, SMX, TET, TMP | *mcr-1* |  |
| 17-SA03201-0 | 2017 | monophasic Typhimurium | pig, gut | pig | AMP, CHL, COL, SMX, TMP | *mcr-1* |  |
| 18-SA00117-0 | 2018 | monophasic Typhimurium | pig | pig | AMP, CHL, COL, SMX, TET, TMP | *mcr-1* |  |
| 18-SA00547-0 | 2018 | monophasic Typhimurium | pork | pork | AMP, COL, SMX, TET | *mcr-1* |  |
| 18-SA00649-0 | 2018 | monophasic Typhimurium | pig, feces | pig | AMP, COL, SMX, TET, TMP | *mcr-1* |  |
| 18-SA00653-0 | 2018 | Typhimurium | pig, feces | pig | AMP, COL, SMX, TET, TMP | *mcr-1* |  |
| 18-SA00907-0 | 2018 | monophasic Typhimurium | piglet | pig | AMP, CHL, COL, SMX, TET, TGC | *mcr-1* |  |
| 18-SA01022-0 | 2018 | Typhimurium | pig, feces | pig | AMP, COL, SMX, TET, TMP | *mcr-1* |  |
| 18-SA01469-0 | 2018 | Typhimurium | pig, organs | pig | AMP, COL, SMX, TET, TGC, TMP | *mcr-4* |  |
| 18-SA01512-0 | 2018 | monophasic Typhimurium | pig | pig | AMP, COL, SMX, TET | *mcr-1* |  |
| 18-SA01513-0 | 2018 | monophasic Typhimurium | pig | pig | AMP, CHL, COL, TET | *mcr-1* |  |
| 18-SA01592-0 | 2018 | monophasic Typhimurium | piglet | pig | AMP, COL, SMX, TET, TMP | *mcr-1* |  |
| 18-SA01610-0 | 2018 | monophasic Typhimurium | pig organs | pig | AMP, COL, GEN, SMX, TET, TMP | *mcr-1* |  |
| 18-SA01665-0 | 2018 | Heidelberg | broiler meat | poultry meat | AMP, CIP, COL, **FOT**, NAL, SMX, **TAZ**, TET, TGC | *mcr-1* | SAMEA5737706 (*bla*_CMY-2_) |
| 18-SA01761-0 | 2018 | monophasic Typhimurium | pig | pig | AMP, COL, SMX, TET, TMP | *mcr-1* |  |
| 18-SA01794-0 | 2018 | monophasic Typhimurium | cat, feces | pet | AMP, COL, SMX, TET | *mcr-1* |  |
| 18-SA01855-0 | 2018 | monophasic Typhimurium | pig, sock swabs | pig | AMP, COL, SMX, TET | *mcr-1* |  |
| 18-SA01865-0 | 2018 | Typhimurium | pig, feces | pig | AMP, CHL, COL, SMX, TET, TGC | *mcr-1* |  |
| 18-SA02206-0 | 2018 | monophasic Typhimurium | pig | pig | AMP, COL, GEN, SMX, TET, TGC, TMP | *mcr-1* |  |
| 18-SA02383-0 | 2018 | monophasic Typhimurium | beef | beef | AMP, COL, SMX, TET, TGC | *mcr-1* |  |
| 18-SA02479-0 | 2018 | Typhimurium | piglet, feces | pig | AMP, CHL, COL, SMX, TET, TGC | *mcr-1* |  |
| 18-SA02480-0 | 2018 | Typhimurium | piglet, feces | pig | AMP, COL, SMX, TET, TGC | *mcr-1* |  |
| 18-SA02517-0 | 2018 | monophasic Typhimurium | pig | pig | AMP, CIP, COL, NAL, SMX, TET | *mcr-1* |  |
| 18-SA03024-0 | 2018 | monophasic Typhimurium | piglet, organs | pig | AMP, COL, GEN, SMX, TET | *mcr-1* |  |
| 18-SA03205-0 | 2018 | monophasic Typhimurium | pig | pig | AMP, COL, TET | *mcr-1* |  |
| 18-SA03301-0 | 2018 | Typhimurium | piglet, organs | pig | AMP, COL, SMX, TET, TMP | *mcr-4* |  |
| 18-SA03305-0 | 2018 | Typhimurium | piglet, organs | pig | AMP, COL, SMX, TET | *mcr-1* |  |
| 18-SA03592-0 | 2018 | monophasic Typhimurium | pig, organs | pig | AMP, COL, SMX, TET | *mcr-1* |  |
