# Supplementary material for: Development of a Novel mcr-6 to mcr-9 Multiplex PCR and Assessment of mcr-1 to mcr-9 Occurrence in Colistin-Resistant Salmonella enterica Isolates From Environment, Feed, Animals and Food (2011–2018) in Germany
Source: Front Microbiol. 2020 Feb 4;11:80. doi: 10.3389/fmicb.2020.00080 (PMC7011100; doi:10.3389/fmicb.2020.00080)
Supplement: Supplementary file 2 [file Table_2.DOCX]

Supplementary Material

# Supplementary Table S2: Colistin-resistant *mcr*-harbouring *Salmonella* isolates sequenced in this study. MLST2.0, ResFinder3.2 and PlasmidFinder2.1 provided at the website of the Center of Genomic Epidemiology (https://cge.cbs.dtu.dk/services/) were used to further analyze the assembled sequencing data. When resistance genes/plasmid markers showed an identity <100 or were shorter than the respective database entries, a “-like” was added to the resistance gene/plasmid marker name. If several resistance genes were assigned to one genetic region, the corresponding names were separated by a “/”.

| **Sample ID** | **BioSample** | **MLST2.0** | **ResFinder3.2** | **PlasmidFinder2.1** |
| --- | --- | --- | --- | --- |
| 11-02308-0 | SAMEA5737697 | 34 | *sul1*, *sul2*, *mcr-1.1*, *bla*_TEM-1B_, *dfrA1*-like, *tet(A)*, *aac(6')-Iaa*, *aadA1*, *aph(3'')-Ib*, *aph(6)-Id* | Col8282, IncFIB(AP001918)-like, IncFII(29)-like, IncQ1-like, IncX4 |
| 11-02585-0 | SAMEA5737699 | 329 | *mcr-4.3*, *aac(6')-Iaa*-like, *parC* p.T57S | - |
| 12-00414-0 | SAMEA5737700 | 27 | *sul1*, *mcr-1.1*, *bla*_TEM-1B_, *aac(6')-Iaa*-like, *aadA2*, *ant(2'')-Ia*, *gyrA* p.S83F | IncX4 |
| 12-01512-0 | SAMEA5737701 | 28 | *sul2*, *sul3*, *mcr-1.1*, *bla*_TEM-1B_, *cmlA1*-like, *dfrA1*, *tet(A)*, *aac(6')-Iaa*-like, *aadA1*, *aadA2b*, *gyrA* p.D87G | IncI1-like, IncX4 |
| 13-SA00018-0 | SAMEA104398375 | 34 | *sul1*, *sul2*, *mcr-1.1*, *mcr-9*-like, *bla*_SHV-12_, *bla*_TEM-1B_, *dfrA19*, *ere(A)*-like, *tet(B)*, *tet(D)*, *aac(6')-IIc*, *aac(6')-Iaa*, *aac(6')-Ib3*/*aac(6')-Ib-cr*-like, *aadA2b*-like, *aph(3'')-Ib*, *aph(3')-Ia*, *aph(6)-Id*, *qnrB2* | IncHI2, IncHI2A, IncQ1-like, IncX4 |
| 13-SA00569-0 | SAMEA5192222 | 34 | *sul1*, *sul2*, *mcr-5.1*, *bla*_TEM-1B_, *tet(A)*, *dfrA1*-like, *aac(6')-Iaa*, *aadA1*, *aph(3'')-Ib*, *aph(6)-Id* | IncFIB(AP001918)-like, IncFII(29)-like, IncQ1-like |
| 13-SA01136-0 | SAMEA5737702 | 34 | *sul2*, *mcr-1.1*, *bla*_TEM-1B_, *tet(B)*, *aac(6')-Iaa*, *aph(3'')-Ib*, *aph(6)-Id* | IncQ1-like, IncX4 |
| 13-SA02656-0 | SAMEA104396046 | 28 | *mcr-1.1*, *mcr-9*-like,*dfrA1*, *aac(6')-Iaa*, *aadA1*-like/*aadA22*-like/*aadA24*-like, *gyrA* p.D87G | IncHI2, IncHI2A, IncI1, IncX4 |
| 13-SA02717-0 | SAMEA5192223 | 34 | *sul2*, *mcr-5.1*, *bla*_TEM-1B_, *dfrA1*, *aac(6')-Iaa*, *aadA1*, *aph(3'')-Ib*, *aph(6)-Id* | IncQ1-like, IncX1-like |
| 14-SA00169-0 | SAMEA104398377 | 34 | *sul1*, *sul2*-like, *mcr-1.1*, *mcr-9*-like, *bla*_SHV-12_, *dfrA19*, *ere(A)*-like, *tet(B)*, *tet(D)*, *aac(6')-IIc*, *aac(6')-Iaa*, *aac(6')-Ib3*/*aac(6')-Ib-cr*-like, *aadA2b*-like, *aph(3'')-Ib*, aph(3')-Ia, *aph(6)-Id*, *qnrB2* | IncHI2, IncHI2A, IncQ1-like, IncX4 |
| 14-SA00170-0 | SAMEA104398378 | 34 | *sul1*, *sul2-like*, *mcr-1.1*, *mcr-9-like*, *bla*_SHV-12_, *bla*_TEM-1B_, *dfrA19*, *ere(A)*-like, *tet(B)*, *tet(D)*, *aac(6')-IIc*, *aac(6')-Iaa*, *aac(6')-Ib3*/*aac(6')-Ib-cr*-like, *aadA2b*-like, *aph(3'')-Ib*, *aph(3')-Ia*, *aph(6)-Id* | IncHI2, IncHI2A, IncQ1-like, IncX4 |
| 14-SA00918-0 | SAMEA104396051 | 34 | *mcr-1.1*, *mcr-9*-like, *dfrA1*, *aac(6')-Iaa*-like, *aadA1*, *gyrA* p.D87G | IncHI2, IncHI2A |
| 14-SA02325-0 | SAMEA5757422 | 34 | *sul1*, *sul2*-like, *mcr-1.1*, *mcr-9*-like, *bla*_SHV-12_, *bla*_TEM-1B_, *dfrA19*, *ere(A)*-like, *tet(B)*, *tet(D)*, *aac(6')-IIc*, *aac(6')-Iaa*, *aac(6')-Ib3*/*aac(6')-Ib-cr*-like, *aadA2b*-like, *aph(3'')-Ib*-like, *aph(3')-Ia*, *aph(6)-Id* | IncHI2, IncHI2A, IncQ1-like, IncX4 |
| 15-SA01381-0 | SAMEA5737695 | 34 | *sul1*, *sul2*, *mcr-1.1*, *mcr-9*-like, *bla*_SHV-12_, *bla*_TEM-1B_, *dfrA19*, *ere(A)*-like, *tet(B)*, *tet(D)*, *aac(6')-IIc*, *aac(6')-Iaa*, *aadA2b*-like, *aph(3'')-Ib*-like, *aph(3')-Ia*, *aph(6)-Id* | IncHI2, IncHI2A, IncX4 |
| 15-SA01864-0 | SAMEA5737696 | 34 | *sul1*, *sul2*-like, *mcr-1.1*, *mcr-9*-like, *bla*_SHV-12_, *bla*_TEM-1B_, *dfrA19*, *tet(B)*, *tet(D)*, *aac(6')-IIc*, *aac(6')-Iaa*, *aac(6')-Ib3*/*aac(6')-Ib-cr*-like, *aadA2b*-like, *aph(3'')-Ib*-like, *aph(3')-Ia*, *aph(6)-Id* | IncHI2, IncHI2A, IncQ1-like, IncX4 |
| 15-SA02829-0 | SAMEA104396062 | 3663 | *sul2*, *mcr-1.1*, *bla*_TEM-1B_, *dfrA1*, *aac(6')-Iaa*-like, *aadA1*, *gyrA* p.D87G | Col(BS512), ColpVC-like, IncI1-like, IncX4 |
| 16-SA02808-2 | SAMEA5737703 | 3663 | *sul2*, *mcr-1.1*, *bla*_TEM-1B_, *dfrA1*, *aac(6')-Iaa*-like, *aadA1*, *gyrA* p.D87G | Col(BS512), ColpVC-like, IncI1-like, IncX4 |
| 17-SA01385-0 | SAMEA5737704 | 34 | *sul3*, *mcr-1.1*, *bla*_TEM-1B_, *cmlA1*-like, *mef(B)*, *dfrA12*, *tet(B)*, *tet(M)*-like, *aac(6')-Iaa*, *aadA1*, *aadA2*-like, *aph(3')-Ia* | IncFIB(AP001918)-like, IncHI2, IncHI2A-like |
| 17-SA02555-0 | SAMEA5737705 | 15 | *sul2*, *mcr-1.1*-like, *bla*_CMY-2_, *fosA7*, *tet(A)*, *aac(6')-Iaa*-like, *parC* p.T57S, *gyrA* p.S83F | IncA/C2, IncX1-like, IncX4 |
| 18-SA01665-0 | SAMEA5737706 | 15 | *sul2*, *mcr-1.1*-like, *bla*_CMY-2_, *fosA7*, *tet(A)*, *aac(6')-Iaa*-like, *aph(3')-Ia*, *parC* p.T57S, *gyrA* p.S83F | ColpVC-like, IncA/C2, IncX1-like, IncX4 |
