# Supplementary material for: Development of a Novel mcr-6 to mcr-9 Multiplex PCR and Assessment of mcr-1 to mcr-9 Occurrence in Colistin-Resistant Salmonella enterica Isolates From Environment, Feed, Animals and Food (2011–2018) in Germany
Source: Front Microbiol. 2020 Feb 4;11:80. doi: 10.3389/fmicb.2020.00080 (PMC7011100; doi:10.3389/fmicb.2020.00080)
Supplement: Supplementary file 3 [file Table_3.docx]

Supplementary Material

# Supplementary Table S3: Additional, colistin-susceptible *mcr-9.1*-harbouring *Salmonella* isolates identified in the NRL *Salmonella* WGS database. Isolates labeled with “*” were previously sequenced using PacBio long-read sequencing and closed bacterial chromosome and plasmid sequences are available under the respective BioSample number. MLST2.0, ResFinder3.2 and PlasmidFinder2.1 provided at the website of the Center of Genomic Epidemiology (https://cge.cbs.dtu.dk/services/) were used to further analyze the assembled sequencing data. When resistance genes/plasmid markers showed an identity <100 or were shorter than the respective database entries, a “-like” was added to the resistance gene/plasmid marker name. If several resistance genes were assigned to one genetic region, the corresponding names were separated by a “/”. Abbreviations: AMP: ampicillin, CHL: chloramphenicol, CIP: ciprofloxacin, FFN: florfenicol, FOT: cefotaxime, GEN: gentamicin, KAN: kanamycin, MERO: meropenem, NAL: nalidixic acid, STR: streptomycin, SMX: sulfamethoxazole, TAZ: ceftazidime, TET: tetracycline, TMP: trimethoprim.

| **Sample ID** | **Year** | ***Salmonella* serovar** | **Source** | **Phenotypic resistance profile** | **COL MIC [mg/L]** | **BioSample** |
| --- | --- | --- | --- | --- | --- | --- |
| 12-01002-0 | 2012 | monophasic Typhimurium | pig, feces | AMP, CHL, CIP, FOT, GEN, KAN, SMX, STR, TAZ, TET, TMP | ≤2 | SAMEA104398373 |
| 12-01738-0* | 2012 | Corvallis | bird | AMP, CHL, CIP, FFN, FOT, KAN, SMX, STR, TAZ, TET, TMP | ≤2 | SAMN08685598 |
| 15-SA01028-0* | 2015 | Infantis | minced meat (pork) | AMP, CHL, FOT, MERO, SMX, TAZ, TMP | ≤1 | SAMN08475067 |
| 16-SA00749-0 | 2016 | Infantis | piglet | AMP, CHL, FOT, SMX, TAZ, TMP | ≤1 | SAMEA4668128 |
| 16-SA01860-0 | 2016 | monophasic Typhimurium | minced meat (beef) | AMP, SMX, TET, TMP | ≤1 | SAMEA6058227 |
| 16-SA02825-0 | 2016 | monophasic Typhimurium | minced meat (pork) | AMP, SMX, TET, TMP | ≤1 | SAMEA104407242 |
| 18-SA00377-0 | 2018 | Agona | food additive | AMP, CHL, CIP, COL, FOT, GEN, NAL, SMX, TAZ, TET, TMP | ≤1 | SAMEA6057867 |
| 18-SA00676-0 | 2018 | Agona | environment | sensitive | ≤1 | SAMEA6058041 |

| **Sample ID** | **SISTR Serovar** | **MLST2.0** | **ResFinder3.2** | **PlasmidFinder2.1** |
| --- | --- | --- | --- | --- |
| 12-01002-0 | 4,[5],12:i:- | 34 | \| *sul1*, *sul2*-like, *mcr-9*, *bla*_SHV-12_, *bla*_TEM-1B_, *catA2*-like, *ere(A)*-like, *dfrA19*, *tet(B)*, *tet(D)*, *aac(6')-IIc*, *aac(6')-Iaa*, *aadA2b*-like, *aph(3'')-Ib*-like, *aph(3')-Ia*, *aph(6)-Id*, *qnrB2* \|  \| \| --- \| --- \| | IncHI2, IncHI2A, IncQ1-like |
| 12-01738-0* | Corvallis | 1541 | *sul1*, *sul2*, *mcr-9*, *bla*_CMY-16_-like/*bla*_CMY-4_-like, *bla*_NDM-1_, *floR*-like, *erm(B)*-like, *mph(E)*, *msr(E)*, *tet(A)*, *fosA3*, *aac(6')-Iaa*-like, *aac(6')-Ib-Hangzhou*-like/*aac(6')-Ib-Suzhou*-like/ *aac(6')-Ib-cr*-like, *aac(6')-Ib3*/*aac(6')-Ib-cr*-like, *aph(3'')-Ib*, *aph(3')-VI*, *aph(3')-VIa*-like, *aph(6)-Id*, *qnrS1*, *parC* p.T57S | IncA/C2, IncHI2, IncHI2A |
| 15-SA01028-0* | Infantis | 32 | *sul1*, *mcr-9*, *bla*_ACC-1_, *bla*_VIM-1_, *catA1*-like, *ere(A)*-like, *aac(6')-Iaa*-like, *aac(6')-Ib3*-like/*aac(6')-Ib-cr*-like, *aadA1*, *aadA1b*, *aph(3'')-Ib*-like, *aph(6)-Id*, *parC* p.T57S | IncHI2, IncHI2A |
| 16-SA00749-0 | Infantis | 32 | *sul1*-like, *mcr-9*, *bla*_ACC-1_, *bla*_VIM-1_, *catA1*-like, *ere(A)*-like, *aac(6')-Iaa*-like, *aac(6')-Ib3*-like/*aac(6')-Ib-cr*-like, *aadA1b*, *aph(3'')-Ib*-like, *aph(6)-Id*, *parC* p.T57S | IncHI2, IncHI2A |
| 16-SA01860-0 | 4,[5],12:i:- | 34 | *sul1*, *sul2*, *mcr-9*, *bla*_TEM-1B_, *mph(B)*, *dfrA1*-like, *tet(A)*, *aac(6')-Iaa*, *aadA1*, *aph(3'')-Ib*, *aph(6)-Id* | IncHI2, IncHI2A, IncQ1-like |
| 16-SA02825-0 | 4,[5],12:i:- | 34 | *sul1*, *sul2*, *mcr-9*, *bla*_TEM-1B_, *mph(A)*, *dfrA19*-like, *tet(B)*, *aac(6')-IIa*-like, *aac(6')-Iaa*, *aadA1*-like, *aadA2b*-like, *aph(3'')-Ib*, *aph(6)-Id* | IncHI2, IncHI2A, IncQ1-like |
| 18-SA00377-0 | Agona | 13 | *sul1*, *sul2*, *mcr-9*, *bla*_SHV-12_, *bla*_TEM-1B_, *catA2*-like, *floR*-like, *ere(A)*-like, *dfrA19*, *tet(D)*, *fosA7*-like, *aac(3)-IIa*, *aac(6')-IIc*, *aac(6')-Iaa*-like, *aadA2b*-like, *aph(3'')-Ib*-like, *aph(3')-Ia*, *aph(6)-Id*, *qnrS1*, *parC* p.T57S, *gyrA* p.S83F | IncHI2, IncHI2A, IncX3, p0111-like |
| 18-SA00676-0 | Agona | 13 | *mcr-9*, *fosA7*-like, *aac(6')-Iaa*-like, *parC* p.T57S | IncHI2, IncHI2A |
